# Upper and Lower Limb Anomalies in Craniofacial Microsomia and Its Relation to the OMENS+ Classification: A Multicenter Study of 688 Patients

**DOI:** 10.1097/PRS.0000000000010090

**Published:** 2022-12-21

**Authors:** Ruben W. Renkema, Thymen Houwen, Christianne A. van Nieuwenhoven, Bonnie L. Padwa, Christopher R. Forrest, David J. Dunaway, Maarten J. Koudstaal, Cornelia J. J. M. Caron

**Affiliations:** Rotterdam, the Netherlands; Boston, MA; Toronto, Ontario, Canada; and London, United Kingdom; From the 1 Dutch Craniofacial Center, Department of Oral and Maxillofacial Surgery; 2 Department of Plastic and Reconstructive Surgery, Erasmus University Medical Center, Sophia’s Children’s Hospital; 3 Craniofacial Centre, Boston Children’s Hospital; 4 Craniofacial Centre, the Hospital for Sick Kids; 5 Craniofacial Unit, Great Ormond Street Hospital.

## Abstract

Craniofacial microsomia (CFM) is characterized by several malformations related to the first and second pharyngeal arch. Patients typically present with facial asymmetry, but extracraniofacial organ systems might be involved, including limb anomalies. The purpose of this study was to analyze the occurrence of upper and lower limb anomalies in CFM patients. Furthermore, the relation between limb anomalies and the OMENS+ (orbital distortion; mandibular hypoplasia; ear anomaly; nerve involvement; soft-tissue deficiency; and associated extracraniofacial anomalies) classification was examined.

**Methods:**

A retrospective study was conducted including patients with CFM from craniofacial units in three different countries. Patients were included when clinical and/or radiographic images were available. Demographic, radiographic, and clinical information was obtained.

**Results:**

A cohort of 688 patients was available and selected for analysis. In total, 18.2% of the patients were diagnosed with at least one upper and/or lower limb anomaly. Upper and lower limb anomalies were seen in, respectively, 13.4% and 7.8% of patients. Patients with other extracraniofacial anomalies had a significantly higher risk for limb anomalies (OR, 27.98; *P* = 0.005). Laterality of CFM and a higher OMENS score were not associated with limb anomalies.

**Conclusions:**

More than one in six patients with craniofacial microsomia have limb anomalies. Therefore, clinical awareness for these anomalies is warranted. Examination and, if present, follow-up on limb abnormalities in patients with CFM should be implemented in the standard assessment of CFM patients.

**CLINICAL QUESTION/LEVEL OF EVIDENCE:**

Risk, III.

Craniofacial microsomia (CFM) is, following cleft lip and palate, the second most frequent congenital disorder of the head and neck. It is estimated to occur in one in 3000 to one in 5000 newborns.^1–3^ CFM is the general term for hypoplasia of facial structures related to the first and second pharyngeal arches.^3–5^ The main characteristics of CFM, resulting in facial asymmetry, include maxillary and/or mandibular hypoplasia, soft-tissue deficiencies, orbital anomalies, preauricular and/or facial tags, and ear anomalies.^2–6^ Wide phenotypic variability resulted in several terms proposed for CFM, including hemifacial microsomia, Goldenhar syndrome, oculoauriculovertebral spectrum, first and second branchial arch syndrome, otomandibular dysostosis, facioauriculovertebral syndrome, and lateral facial dysplasia.^3,6,7^

Several classification systems have been developed for CFM. The most well-known and well-used classifications are the Pruzansky-Kaban classification and the OMENS+ classification.^8–10^ The Pruzansky-Kaban classification is used to score the severity of mandibular hypoplasia. The OMENS+ classification documents anomalies of the orbit, mandible, ear, nerve function, and soft-tissue deficiencies. The “plus” is used for the expanded spectrum with respect to extracraniofacial anomalies.^7^

Extracraniofacial anomalies might be present in the vertebrae, central nervous system, circulatory tract, gastrointestinal tract, and/or urogenital tract.^6,7,11,12^ Analyses of the presence of these anomalies in patients with CFM indicated that they are correlated with more severely affected facial phenotypes.^7,12^

Early studies have documented limb anomalies in patients with CFM.^7,13–20^ In the general population, upper limb anomalies are estimated to be present in 11.4 to 19.7 (0.001 to 0.002%) per 10,000 newborns.^21–23^ Some anomalies such as hip dysplasia/dislocation occur more frequently in girls, whereas others such as clubfoot and polydactyly occur more frequently in boys.^24^ Limb anomalies might be more prevalent in patients with CFM. Horgan et al. described in a population of 121 patients with CFM that 41% were diagnosed with skeletal anomalies. This included both limb and nonlimb skeletal anomalies.^7^ Other studies by Werler et al. and Beleza-Meireles et al. included, respectively, 239 and 51 patients with CFM and described anomalies of the limbs in 7% to 12% of the patients.^19,20^

A variety of limb anomalies were described, including radial dysplasia, thumb hypoplasia, scaphoid aplasia,^14–16,18^ clubfoot, congenital hip dislocation, Sprengel deformity,^7,15^ preaxial polydactyly,^7,16^ and finger anomalies.^7^ Even though the previous literature described the presence of limb anomalies in patients with CFM, research with detailed data on limb anomalies in a larger cohort has not been performed.

The purpose of this study was to analyze the occurrence of upper and lower limb anomalies in patients with CFM, by studying the type and prevalence of these anomalies. Second, we aim to determine whether there is an association between the phenotypic severity of CFM and the presence of limb anomalies. We hypothesize that, in accordance with other extracraniofacial anomalies, limb anomalies are more frequently present in patients with CFM compared with the general population and more frequently seen in patients with severe facial hypoplasia and/or the “expanded spectrum” of CFM.^12^

## PATIENTS AND METHODS

This retrospective study was conducted in the population of patients diagnosed with CFM at the craniofacial centers of the Erasmus University Hospital, Rotterdam, The Netherlands; the Great Ormond Street Hospital, London, United Kingdom; and the Hospital for Sick Kids, Toronto, Ontario, Canada.

Following institutional review board approval (Rotterdam, MEC-2012-248; London, 14 DS25; and Toronto, 1000053298), the medical files of all patients diagnosed with CFM were reviewed. Although microtia might be seen as a mild phenotype of CFM, patients with microtia as an isolated anomaly were excluded from further analyses in this study.

CFM is a clinical diagnosis based on physical examination and examination of radiographic images. Therefore, only patients with panoramic radiographs, computed tomographic scans of the head, and/or available clinical photographs supporting the diagnosis CFM were included. All medical charts of patients meeting our inclusion criteria were searched for date of birth, sex, affected side, presence of extremity anomalies, treatment of extremity anomalies, and available clinical photographs.

The severity of CFM was scored in patients using the Pruzansky-Kaban classification and the OMENS+ classification.^7,25^ For bilateral cases, the most severely affected side was used for descriptive and statistical analyses. Patients unable to be classified with the Pruzansky-Kaban classification were graded as unknown. This group was excluded from further statistical analysis and only used for descriptive statistics. Extracraniofacial anomalies included vertebral, cardiac, central nervous system, renal, gastrointestinal, and respiratory anomalies. Limb anomalies were categorized as a separate entity.

Limb anomalies were considered as congenital aberrations of arms and/or legs from the proximal shoulder or hip joint to the distal end of the limbs (ie, from shoulder to fingertip and from hip to toes). The Blauth classification (Blauth types I, II, IIIA, IIIB, IV, and V) was used for scoring severity of thumb hypoplasia. Blauth type I is present when only minor hypoplasia is seen, Blauth type II shows metacarpophalangeal joint instability and thenar hypoplasia, Blauth type III is characterized by musculotendinous and osseous deficiencies, Blauth type IV is the floating thumb, and Blauth type V is total absence of the thumb.^26^

Because patients could be affected with multiple different observational extremity anomalies, each classifiable limb anomaly was recorded and counted as one separate anomaly. As an example, bilateral cases and multiple unilateral cases of anomalies were counted as individual problems. The total number of separate identifiable limb anomalies was therefore higher than the total number of patients with any limb anomaly.

### Statistical Analysis

Statistical analyses were performed using IBM SPSS Statistics for Windows, version 24.0 (IBM Corp., Armonk, NY). Descriptive analyses were initially performed. A chi-square test was used to assess the correlation between sex, laterality of CFM and extracraniofacial anomalies, and the presence of limb anomalies. The Fisher exact test was used if the assumptions for Pearson chi-square test were violated (ie, expected count less than five). The correlation between the affected facial and limb side was studied, in which patients with unilateral CFM and bilateral limb anomalies were considered to have limb anomalies on the contralateral side. Analyses were repeated after exclusion of patients with isolated clinodactyly, isolated camptodactyly, or isolated trigger thumb, as these anomalies are also regularly seen in nonsyndromic persons. The association between the OMENS+ categories and limb anomalies were assessed by univariable and multivariable binary logistic regression analysis. This was expressed by odd ratios, 95% confidence intervals, and *P* values. All statistical tests used a two-sided significance level of 0.05. Goodness of model fit was based on the model chi-square (*P* value). Multicollinearity (correlations within all components of the model) was examined. The discriminative ability of the multivariable logistic regression model was validated by a receiver operating characteristic curve.

## RESULTS

### Study Characteristics

A total cohort of 688 patients was available for analysis. The patient characteristics are shown in Table 1. Unilateral CFM was seen in 615 patients (89%) and bilateral CFM in 73 patients (11%). Slightly more male patients (*n* = 367) than female patients (*n* = 321) were included.

**Table 1.** Description of the Population

	Limb Anomalies				*P*
	Yes		No		
	No.	%	No.	%	
Total	125	18	563	82	
Sex					
Male	65	18	302	82	0.74
Female	60	19	261	81	
Laterality					
Unilateral, right	58	17	280	83	0.23
Unilateral, left	50	18	227	82	
Bilateral	17	24	55	76	
Orbit					
0	28	14	168	86	0.27
1	21	22	74	78	
2	17	18	76	82	
3	15	17	74	83	
4	9	24	29	76	
U/A	34	20	137	80	
Mandible					
1	22	15	122	85	0.49
2A	41	26	119	74	
2B	14	13	91	87	
3	16	15	88	85	
U/A	32	18	143	82	
Ear					
0	11	19	48	81	0.50
1	14	17	70	83	
2	20	32	43	68	
3	37	15	206	85	
4	4	18	18	82	
U/A	38	18	173	82	
Nerve					
0	11	14	67	86	0.78
1	1	11	8	89	
2	2	20	8	80	
3	1	20	4	80	
4	3	15	17	85	
U/A	106	19	454	81	
Soft tissue					
0	8	19	35	81	0.19
1	31	14	189	86	
2	38	20	149	80	
3	12	21	44	79	
U/A	35	20	141	80	
Extracraniofacial					
Yes	77	28	197	72	<0.001^a^
No	23	7	283	93	

U/A, unavailable data.

a Statistically significant.

### Presence of Limb Anomalies

In total, 18.2% (*n* = 125) of the patients were diagnosed with at least one anomaly of the upper and/or lower limbs (Table 1). Limb anomalies were observed in 17.6% of the patients with unilateral CFM and in 23.3% of the patients with bilateral CFM. There was no statistical difference in the prevalence of limb anomalies in unilateral versus bilateral CFM [Pearson chi-square (1, *n* = 688) = 1.4; *P* = 0.23].

**Fig 1.**
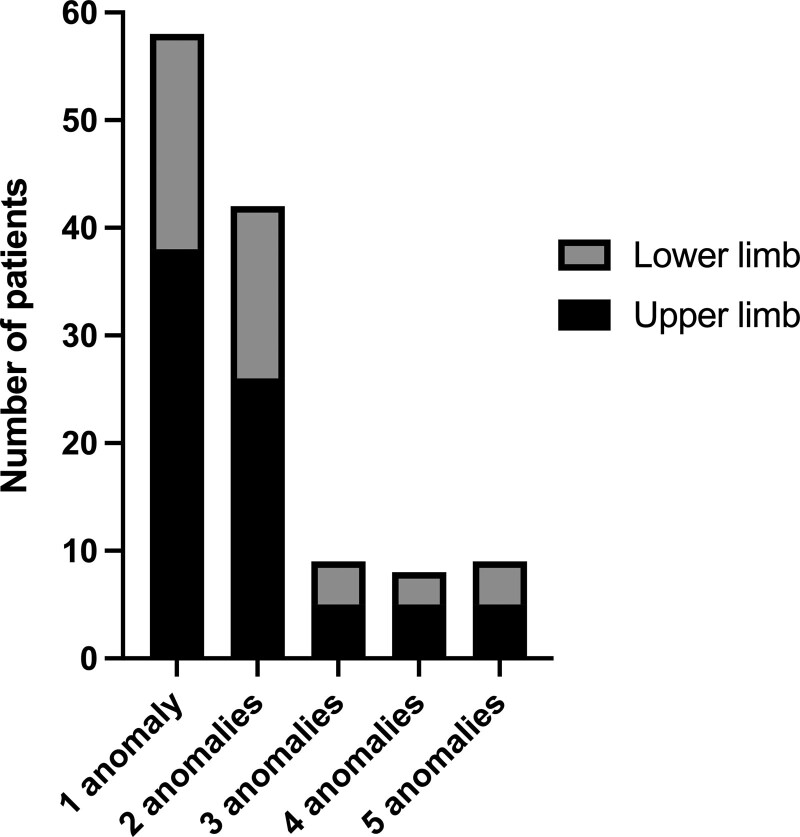
Number of extremity anomalies per patient.

Fifty-seven patients (46%) had one limb anomaly and 68 patients (54%) had multiple anomalies of the upper and/or lower limb (Fig. 1). Most patients (57%) had anomalies of the upper limbs, 26% had anomalies of the lower limbs, and 17% of the patients had both upper and lower limb anomalies.

### Upper Limb Anomalies

There were 92 patients (13.4%) with upper limb anomalies. This included both unilateral and bilateral limb involvement with a wide spectrum of anomalies.

Radial ray deficiencies and abnormalities were observed in 78 cases including thumb hypoplasia, thumb in palm, triphalangeal thumb, thumb duplication, and radial dysplasia. Table 2 shows the individual numbers of all upper limb anomalies. Examples of observed radial ray anomalies in our cohort are presented as Supplemental Digital Content. (**See Figure, Supplemental Digital Content 1**, which shows Blauth type IIIB thumb hypoplasia, convergent polydactyly Wassel type IV, Wassel type IV thumb duplication, radial dysplasia type III with absent thumb, and radiograph of right-side radial cleft hand, http://links.lww.com/PRS/F783.)

**Table 2.** Type of Upper Limb Anomalies

Upper Limb Anomaly	Frequency of Occurrence (%)	No. of Patients Affected (%)
Total	159	92
Thumb hypoplasia		
Blauth type I	7 (4.4)	6 (6.5)
Blauth type II	11 (6.9)	11 (12.0)
Blauth type IIIA	3 (1.9)	3 (3.3)
Blauth type IIIB	5 (3.1)	3 (3.3)
Blauth type IV	5 (3.1)	5 (5.4)
Blauth type V	10 (6.3)	9 (9.8)
Unspecified	16 (10.1)	10 (10.9)
Thumb in palm	2 (1.3)	2 (2.2)
Triphalangeal thumb	3 (1.9)	3 (3.3)
Thumb duplication	9 (5.7)	8 (8.7)
Radial dysplasia	22 (13.8)	18 (19.6)
Brachydactyly	9 (5.7)	6 (6.5)
Camptodactyly	4 (2.5)	3 (3.3)
Clinodactyly	21 (13.2)	13 (14.1)
Syndactyly	11 (6.9)	10 (10.9)
Central polydactyly	1 (0.6)	1 (1.1)
Broad thumb	6 (3.8)	3 (3.3)
Cleft hand	1 (0.6)	1 (1.1)
Congenital scaphoid malformation	1 (0.6)	1 (1.1)
Ulna hypoplasia	1 (0.6)	1 (1.1)
Hemihypoplasia of upper limb not specified	4 (2.5)	4 (4.3)
Rudimentary clavicle	1 (0.6)	1 (1.1)
Sprengel deformity	4 (2.5)	3 (3.3)
Trigger thumb	1 (0.6)	1 (1.1)
Finger aplasia	1 (0.6)	1 (1.1)

Finger abnormalities were the second largest group observed in our cohort and included brachydactyly, camptodactyly, clinodactyly, syndactyly, and central polydactyly. (Table 2). Less frequent anomalies included cleft hand, Sprengel deformity, abnormal broad thumbs, hemihypoplasias of the upper limb, trigger thumbs, congenital scaphoid malformation, finger aplasia, ulna hypoplasia, and a rudimentary clavicle. The number of patients affected with these anomalies varied from one to four (Table 2).

Sidedness of upper limb anomalies compared with the hypoplastic facial side is shown in Table 3. Although not significant, anomalies of the upper extremities occurred more frequently on the same side as the CFM-affected side [Pearson chi-square (1, *n* = 75) = 0.04; *P* = 0.85]. This was the case in 46 of the 75 patients (61.3%) with unilateral CFM and upper extremity anomalies. Exclusion of the 13 patients with isolated clinodactyly, isolated camptodactyly, or isolated trigger thumb had a small effect on the overall prevalence of limb anomalies in patients with CFM (18.2 to 16.6%) and had no effect on any statistical analysis.

**Table 3.** Laterality of CFM and Limb Anomaly^a^,^b^

	Left-Side CFM (%)	Right-Side CFM (%)	Bilateral CFM (%)
Left limb anomaly	22 (44)	1 (2)	3 (18)
Left upper anomaly	12 (24)	0 (0)	3 (18)
Left lower anomaly	5 (10)	1 (2)	0 (0)
Left upper and lower	5 (10)	0 (0)	0 (0)
Right limb anomaly	10 (20)	28 (48)	5 (29)
Right upper anomaly	4 (8)	23 (40)	2 (12)
Right lower anomaly	6 (12)	3 (5)	3 (18)
Right upper and lower	0 (0)	2 (3)	0 (0)
Bilateral limb anomaly	16 (32)	27 (47)	8 (47)
Bilateral upper	6 (12)	13 (22)	4 (24)
Bilateral lower	7 (14)	6 (10)	1 (6)
Bilateral upper and lower	3 (6)	8 (14)	3 (18)
Unknown side anomaly	2 (4)	2 (3)	1 (6)
Unknown upper	2 (4)	1 (2)	1 (6)
Unknown lower	0 (0)	1 (2)	0 (0)
Total	50 (100)	58 (100)	17 (100)

a CFM, in number of affected cases.

b Pearson χ^2^, 0.26; *P* = 0.61.

### Lower Limb Anomalies

There were 54 patients (7.8%) with one or multiple lower limb anomalies. Patients presented with a wide spectrum of problems ranging from hip dislocation or hip dysplasia to feet anomalies. Clubfeet were documented as equinus valgus, talipes equinovarus, calcaneovalgus, talus deformity, or unspecified. The remaining group of anomalies consisted of a range of deformities, including flat feet, hemihypotrophy of the leg, toe deformities, flexion contractures, and metatarsus adductus (Table 4).

**Table 4.** Type of Lower Limb Anomalies

Lower Limb Anomaly	Frequency of Occurrence (%)	No. of Patients Affected (%)
Total	83	54
Clubfoot		
Equinus valgus	7 (8.4)	4 (6.1)
Talipes equinovarus	5 (6.0)	4 (6.1)
Calcaneovalgus	2 (2.4)	1 (1.5)
Talus deformity	2 (2.4)	1 (1.5)
Unspecified	4 (4.8)	2 (3.0)
Flat feet (pes planus/plano valgus)	13 (15.7)	9 (13.6)
Congenital hip dislocation/dysplasia	9 (10.8)	7 (10.6)
Clinodactyly	5 (6.0)	4 (6.1)
Syndactyly	7 (8.4)	7 (10.6)
Flexion contracture	3 (3.6)	3 (4.5)
Hemihypotrophy	7 (8.4)	7 (10.6)
Metatarsus adductus	2 (2.4)	2 (3.0)
Other	14 (16.9)	13 (19.7)
Unspecified	3 (3.6)	2 (3.0)

Sidedness of lower limb anomalies compared with the hypoplastic facial side is shown in Table 3. Anomalies of the lower limbs occurred in 35.7% (*n* = 15) on the same side as the CFM-affected side. Patients were not significantly more affected on the same side with lower limb anomalies as their CFM affected side [Pearson chi-square (1, *n* = 42) = 0.078; *P* = 0.78)]. The laterality of CFM was not correlated with the laterality of the limb anomaly [Pearson chi-square (1, *n* = 101) = 0.26; *P* = 0.61].

### Associated Factors

**Fig 2.**
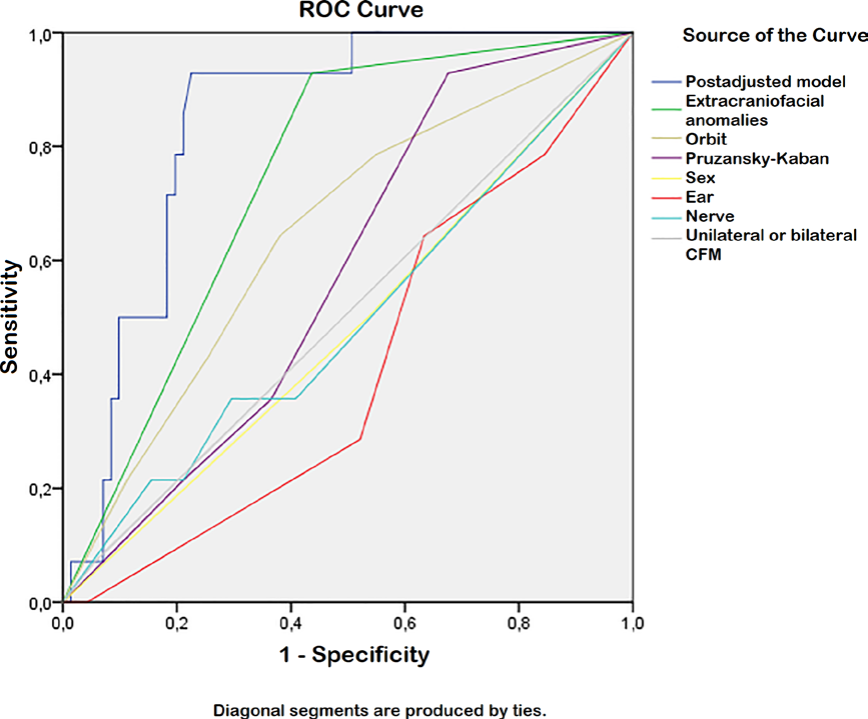
Receiver operating characteristic (ROC) curve.

The odds for having limb anomalies were analyzed by univariable and multivariable postadjusted logistic regressions, as shown in Table 5. The individual OMENS+ categories, sex, and unilaterality or bilaterality of CFM were analyzed separately in logistic regressions. This showed a statistically significant association between the presence of extracraniofacial anomalies and limb anomalies (OR, 4.81; 95% CI, 2.92 to 7.91; *P* < 0.001). Multicollinearity (correlation between sex, laterality, and the individual OMENS+ categories) was checked for the multivariable model, leading to exclusion of the soft-tissue score, as this was correlated to the mandible score (Pearson *R*, 0.24; *P* < 0.001). The final multivariable model showed that the presence of extracraniofacial anomalies was significantly associated with an increased risk for limb anomalies, when adjusted for sex; laterality of CFM; and the orbit, mandible, ear, and nerve score (OR, 27.98; 95% CI, 2.68 to 291.96; *P* = 0.005). The area under the receiver operating characteristic curve was 0.84 (95% CI, 0.75 to 0.93%) (Fig. 2).

**Table 5.** Univariable and Multivariable Logistic Regression Analyses for the Association between Patient Characteristics and Limb Anomalies^a^

	Univariate Logistic Regression		Multivariate Logistic Regression Model	
	OR (95% CI)	*P*	OR (95% CI)	*P*
Constant	—	—	0.035	0.014^b^
Female gender	1.07 (0.73–1.57)	0.74	1.26 (0.31–5.17)	0.75
Bilateral CFM	1.43 (0.80–2.55)	0.23	0.28 (0.02–3.90)	0.34
Orbit score^c^	1.10 (0.93–1.30)	0.27	1.41 (0.86–2.30)	0.18
Mandible score^c^	0.93 (0.75–1.14)	0.49	0.81 (0.40–1.61)	0.55
Ear score^c^	9.94 (0.77–1.14)	0.50	0.64 (0.36–1.16)	0.14
Nerve score^c^	1.05 (0.76–1.44)	0.78	1.02 (0.67–1.55)	0.94
Soft-tissue score^c^	1.21 (0.91–1.61)	0.19	—	—
Extracraniofacial anomalies^c^,^d^	4.81 (2.92–7.91)	<0.001^b^	27.98 (2.68–291.96)	0.005^b^

a Goodness of model fit = 18.47; *P* = 0.01.

b Statistically significant.

c Component of the OMENS+ classification.

d Includes nonlimb extracraniofacial anomalies only.

## DISCUSSION

The purpose of this study was to analyze the occurrence of both upper and lower limb anomalies in patients with CFM. We hypothesized that limb anomalies would occur more than expected in the general population. All limb anomalies were described, including prevalence and type of upper and lower limb anomalies. Furthermore, we aimed to study factors in patients with CFM that might be associated with a higher risk for limb anomalies.

A total of 125 CFM patients (18.2%) in this cohort were diagnosed with anomaly of the upper and/or lower limb. Ninety-two patients (13.4%) had upper limb anomalies that were mainly characterized by malformations of the radioulnar axis as the dominant affected axis; 96 anomalies were observed. Associated problems with the radioulnar axis can be severely impairing because it is crucial for strength and grip in our daily use. Finger anomalies were documented in 33 patients.

Fifty-four patients (7.8%) had lower limb anomalies. Clubfeet were seen in 12 patients (1.7%). The general incidence of clubfoot is approximately 10 per 10,000 live births for the isolated condition. However, clubfeet are frequently seen as part of a skeletal dysplasia or syndrome.^26^ The prevalence of clubfeet in this study is 17 times higher than in the general population.

Analysis of risk factors showed that patients with CFM and extracraniofacial anomalies have a significantly higher risk for limb anomalies (OR, 27.98), adjusted for sex, laterality (unilateral or bilateral CFM), and OMENS severity. The severity of facial hypoplasia (as displayed in the OMENS score) and the presence of bilateral CFM were not associated with limb anomalies. This is interesting, as previous studies on nonlimb extracraniofacial anomalies in CFM showed a higher prevalence of these anomalies among more severely affected patients.^7,11,12^ The correlation between limb anomalies and other extracraniofacial anomalies could suggest a shared pathophysiologic mechanism for patients with the expanded spectrum of CFM. Limbs develop in week 4 of development by formation of limb buds, initiated by undifferentiated mesenchyme and ectodermal covering.^27^ The origin of CFM is yet unknown. It is hypothesized that an error in neural crest cell migration might be responsible for the anomalies observed in patients with CFM.^7,28^ The embryonic origin of limb anomalies in patients with CFM remains unknown.

The prevalence of 18% indicates that extremity anomalies are common in CFM patients next to other extracranial anomalies.^7,11,12^ Upper limb anomalies in the general population occur in 11.4 to 19.7 per 10,000 (one in 877 to one in 508) newborns according to the literature.^21–23^ Comparing the results of our findings with the previous studies shows a prevalence higher than Werler et al. (7% limb anomalies in a studied cohort of 239 patients) and Beleza-Meireles et al. (12% limb anomalies in a studied cohort of 51 patients), but lower than Horgan et al. (20.7% in a studied cohort of 121 patients).^7,19,20^ All three studies especially described radial ray abnormalities, including thumb hypoplasia^7,20^ and preaxial polydactyly.^19,20^ Furthermore, syndactyly and limb reduction defects were described in Werler et al.^19^ Beleza-Meireles et al. showed hip dysplasia to be present in two cases.^20^ The description of, especially, Werler et al. is limited, as only the presence of limb anomalies is described and divided into limb reduction defects and polydactyly or syndactyly without further specification. Several case reports and retrospective studies described especially radial ray problems and other observed extremity anomalies.^7,13,16–18,20^ This retrospective cohort study presents all limb anomalies observed in different categories, by studying a large cohort of patients with CFM. All previously described limb anomalies in CFM were observed in this study too.

### Incorporation in Standard CFM Care

Birgfeld and Heike suggested a standard protocol for CFM in 2012. A surgical and medical treatment timeline is presented for individuals with CFM.^29^ Treatment and evaluations are divided into different age groups, with most endangering and critical triaged (ie, breathing and feeding first). Internal organ assessment for renal problems and cardiac anomalies are a next important step, but simultaneously all other possible anomalies should be examined. The subcategory of limb anomalies should be implemented. As limb anomalies might be minor and difficult to diagnose, evaluation and discussion of potential treatment should take place by experienced (plastic or orthopedic) surgeons shortly after birth. Treatment and rehabilitation can contribute to improved body functions, aimed to increase the manual activity capacity to better perform daily activities. Regular check-ups for manual capacity and foot function is indicated for all patients with limb anomalies.

### Limitations

This study has several limitations. First, no age limit was used to include patients with CFM. This is necessary to avoid selection bias, but also bears the risk of information bias. The retrospective nature of this study increases the risk for incomplete data, which is even higher in older patients. The numbers of extremity anomalies are possibly higher than we have found in our study. A prospective study would be able to determine the precise prevalence of limb anomalies in CFM.

As already discussed in the timeline created by Birgfeld and Heike,^29^ patients with CFM can present with numerous difficulties that might require attention first. Subtle and possible insignificant abnormalities can therefore be missed and not documented in the patient files. This might also have led to an underestimation of the observed limb anomalies. However, knowing that limb anomalies occur in a substantial number of patients with CFM, it is clinically important to document all anomalies to monitor motor skills and progress.

## CONCLUSIONS

More than one in six patients with CFM showed limb anomalies. Patients with other extracraniofacial anomalies are at increased risk for limb anomalies. No correlations between facial phenotype and limb anomalies were found. As a significant number of patients with CFM experience limb anomalies, clinical awareness for these anomalies is warranted.

## DISCLOSURE

The authors have no financial interests or conflicts of interest to report. This study was not funded.

## Acknowledgments

The authors thank Bran Sivakumar, FRCS(Plast), consultant plastic surgeon at Great Ormond Street Hospital and Gill Smith, FRCS(Plast), consultant plastic and reconstructive surgeon at Great Ormond Street Hospital, for knowledge and support. The authors also thank E. E .C. M. Elsten, MD, H. A. Galema, MD, M. J. Schreuder, MD, and J. H. Stokker, MD, research assistants at Erasmus University Hospital, for assistance with the database.

## Supplementary Material


