# Supplementary material for: Upper and Lower Limb Anomalies in Craniofacial Microsomia and Its Relation to the OMENS+ Classification: A Multicenter Study of 688 Patients
Source: Plast Reconstr Surg. 2022 Dec 21;151(5):1053–61. doi: 10.1097/PRS.0000000000010090 (PMC10125112; doi:10.1097/PRS.0000000000010090)

Blauth type IIIB thumb hypoplasia

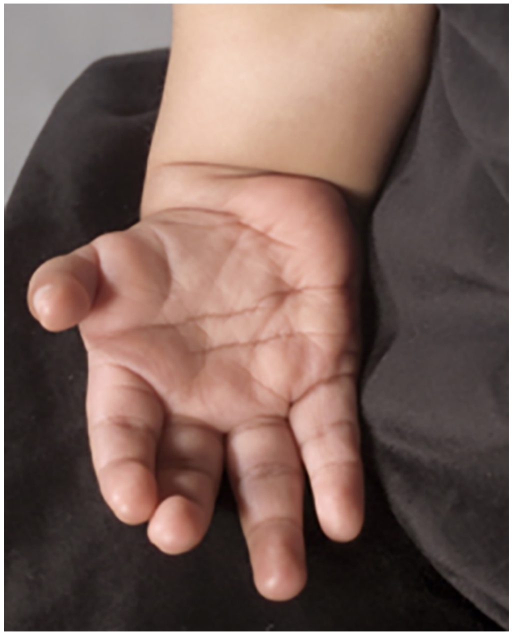

Convergent polydactyly Wassel IV

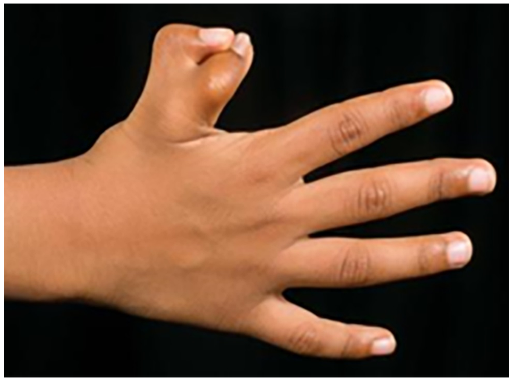

Wassel IV thumb duplication

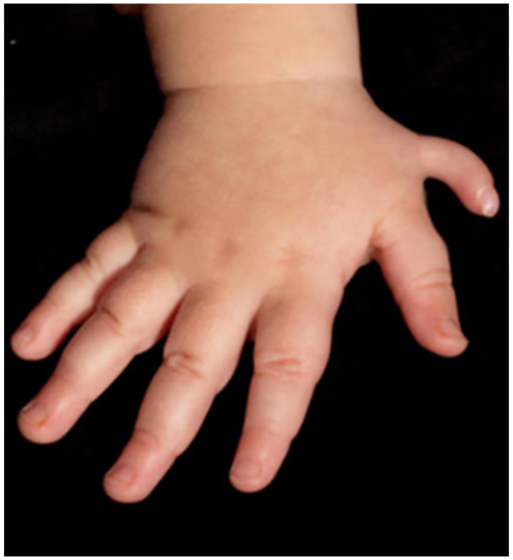

Radial dysplasia type III with absent thumb

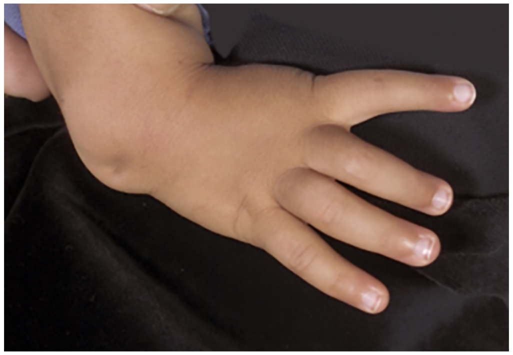

X-ray of right-sided radial cleft hand

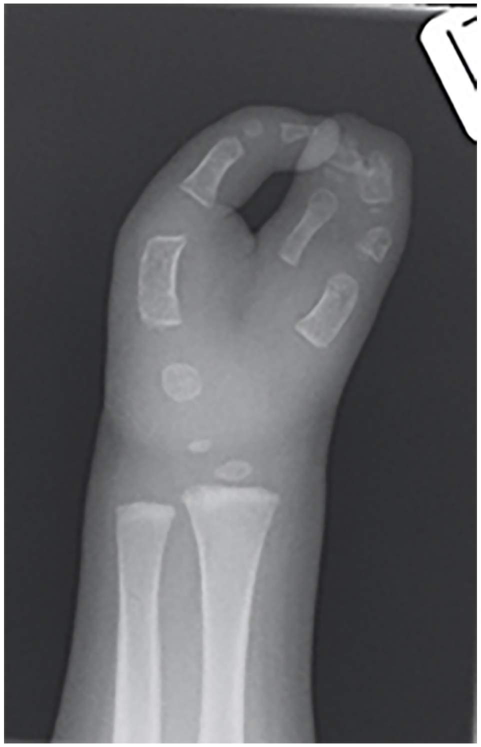

Supplement: Supplementary file 1 [file prs-151-1053-s001.pdf]
